# Quantification of illicit and prescription drug residues in pasteurized donor human milk: a comprehensive analytical approach

**DOI:** 10.1038/s41372-025-02443-6

**Published:** 2025-10-09

**Authors:** Michelle Brenner, Kaytlin Krutsch, Ashlynn Baker, Dhavalkumar Patel, Palika Datta

**Affiliations:** 1 The King’s Daughters Milk Bank, Children’s Hospital of The Kings Daughters, Department of Pediatrics, Macon & Joan Brock Virginia Health Sciences at Old Dominion University, Norfolk, VA USA; 2 Texas Tech University Health Sciences Center InfantRisk Center, Amarillo, TX USA; 3 Department of Pharmaceutical Sciences, Jerry H. Hodge School of Pharmacy, Texas Tech University Health Sciences Center, Amarillo, TX USA

**Keywords:** Paediatrics, Risk factors

## Abstract

**Objective:**

To assess the risk of infant exposure to illicit and prescription drug residues, from infant-ready, pooled, pasteurized donor human milk (PDHM) provided by healthy, screened, voluntary non-remunerated donors to a not-for-profit milk bank.

**Study design:**

A total of 150 pooled samples, representing 742 donor contributions, were analyzed for 15 illicit and prescription drug analytes using liquid chromatography-tandem mass spectrometry (LC-MS/MS). Detected concentrations were evaluated for clinical significance.

**Result:**

All 150 PDHM samples tested negative for residues of marijuana, amphetamines, benzodiazepines, fentanyl, hydrocodone, and morphine. Three samples (2%) contained trace amounts of oxycodone (<0.6 parts per billion), yielding exposures over 455 times lower than established standards for infant safety (Relative Infant Dose: <0.022%; standard safety threshold 10%).

**Conclusion:**

Comprehensive analysis detected no clinically relevant illicit or prescription drug residues in infant-ready pasteurized donor human milk, supporting both its safety for neonatal consumption and the effectiveness of current donor screening protocols and milk processing practices.

## Introduction

Any environmental exposure a lactating woman experiences has the potential to transfer into her breast milk to some extent, potentially exposing the infant consuming milk to residual maternal substances. Simultaneously, pasteurized donor human milk (PDHM) serves as a lifeline for medically fragile infants in neonatal intensive care units (NICUs) when the mother’s own milk is unavailable [1, 2]. Subsequently, concerns have emerged regarding the potential risk of infant exposure to illicit and prescription drugs through PDHM [3, 4]. These concerns have been amplified by two trends: rising rates of substance use in the general population and the increasing potency of illicit substances, particularly synthetic opioids like fentanyl, which can “adulterate” the milk and cause adverse effects at minute concentrations. Though rare, documented cases exist of infant harm following exposure to maternal substances via the mother’s own milk, whether from medically supervised or illicit drug use [5]. For vulnerable NICU infants with immature metabolic and excretory systems, exposures to such residues pose risk, though these potential harms must be carefully weighed against the well-established benefits of human milk over commercial formula for this population. Although no cases of neonatal adverse effects from illicit or prescription drug exposure via PDHM have been reported in the medical literature, a comprehensive risk-benefit assessment requires empirical data quantifying drug adulterant prevalence, rather than reliance on theoretical concerns or reassurance from the absence of observed harm.

Substance use occurs in the lactating population. The prevalence of illicit drug use, specifically among breastfeeding women and milk donors, remains largely unknown, creating a critical knowledge gap. Cannabis is reported among approximately 5% of breastfeeding mothers according to Pregnancy Risk Assessment Monitoring System data from 2017, making it the most likely adulterant drug in human milk [6]. Other substance use documented in breastfeeding populations includes cocaine, opioids, and methamphetamine, all of which can transfer into breast milk as adulterant residues to varying degrees [7–9]. Medically supervised use of these and similar compounds (e.g., opioids, amphetamines) is less frequently posited as a concern, but also confers the same mechanistic risk to the infant.

Not-for-profit milk banks employ multi-layered strategies to mitigate potential risks of illicit and prescription drug residues in milk. Like blood banking [10], the cornerstone of these efforts is rigorous donor screening, which typically includes comprehensive health and medication histories, lifestyle questionnaires, serologic testing, and endorsement from a previously established healthcare provider [11, 12]. Importantly, these banks rely exclusively on voluntary, non-remunerated donors—a model that, again similar to blood banking [13], may attract individuals more committed to providing safe milk compared to paid donation models. Consequently, human milk obtained from carefully screened donors is designed to offer a supply with minimal risk of drug residues, providing a valuable option for medically vulnerable infants without access to a full diet of the mother’s own milk.

Beyond screening, the standard practice of combining milk from multiple donors (“pooling”) yields critical, strategic advantages that are often overlooked [12, 14]. Pooling facilitates standardization of nutritional composition across production batches, creates substantial dilution of any potential adulterants from individual contributors (reducing even theoretically significant concentrations to clinically insignificant levels), and mitigates “outlier effects” where idiosyncratic milk characteristics from a single donor might otherwise dominate. Furthermore, pooling enhances the immunological profile of batches by broadening the spectrum of bioactive components, including diverse antibodies, human milk oligosaccharides, vitamins, and other beneficial constituents essential for optimal infant development [15].

There is growing evidence supporting the effectiveness of these approaches. In a 2014 validation study, Escuder-Vieco et al. tested 400 pooled specimens from 63 donors using liquid chromatography tandem mass spectrometry (LC-MS/MS) and found no presence of caffeine, nicotine, morphine, amphetamines, cocaine, cannabis, methadone, or related metabolites, aligning with responses from donor screening questionnaires [11]. A follow-up study in 2016 confirmed the lack of substance use in 36 donors who reported no episodic or chronic use of the same substances during screening using two methodologies: (1) analysis of donor milk by LC-MS/MS, which indicated no drug use around the time of milk expression, and (2) hair sample testing by gas chromatography-mass spectrometry (GC-MS), which provided a longer retrospective view suggesting no illicit drug exposure during pregnancy and in the early stages of breastfeeding [16]. In their 2023 investigation, Ramos Santos et al used GC-MS to detect cocaine, nicotine, morphine, methadone, amphetamines, and ketamine (and metabolites) in breast milk. Notably, all of their 67 samples (unpooled) from a milk bank tested negative for the entire panel of analytes [17]. These studies each have potential methodological limitations, and the analytes quantified may not capture the most prevalent adulterants expected in our community.

Ideally, milk banks and NICUs would have access to an inexpensive, rapid, accurate, validated, and user-friendly test capable of detecting bioactive adulterant residues directly in milk. Among the available drug testing matrices—blood, breath, saliva, urine, sweat, and hair—only sweat and hair testing can provide a potential indication of drug use occurring more than several weeks prior [18]. In contrast to blood banking, no US Food and Drug Administration (FDA)-approved in vitro diagnostic assays currently exist for adulterant detection in human milk. Current enzyme-linked immunosorbent assay (ELISA) analytical methods for detecting adulterants in human milk are problematic [19]. ELISA assays marketed for milk banking are only internally validated for forensic use rather than any diagnostic purposes [20], presumably as manufacturers have not met FDA standards for validating the assays in the complex matrix of human milk. Further, ELISA testing in human milk typically requires sample processing that may demand specialized expertise or equipment beyond the routine capabilities of many milk banks. Consequently, milk testing practices vary significantly between milk banks with some facilities implementing forensic ELISA analytical milk testing in addition to donor screening protocols.

Given these considerations, a critical clinical question emerges: Under current donor screening protocols and milk processing practices, does infant-ready pooled PDHM contain illicit or prescription drug residues of concern at levels that might pose harm to medically fragile infants? This study addresses this knowledge gap by employing highly sensitive analytical methods to examine a large sample of PDHM from a not-for-profit milk bank accredited by the Human Milk Banking Association of North America (HMBANA) for these residues, generating evidence-based data to inform clinical practice and milk banking policies.

## Materials/subjects and methods

### Study setting and design

This study was conducted at The King’s Daughters Milk Bank (KDMB), one of 32 not-for-profit donor human milk banks in North America accredited by the HMBANA. KDMB collects, processes, and distributes PDHM to medically fragile infants in NICUs, newborn nurseries, and outpatients with medical need on the Eastern seaboard. During processing of 977 batches, from December 2022 to June 2024, a total of 150 random samples of pooled PDHM were collected for drug analyte testing. Pooled samples were aggregated from an average of 5 donors (range of 4–8), with a total of 742 donor contributions in the 150 tested samples. Informed consent was obtained from all donors as part of the standard intake process. This study was approved by the Institutional Review Board and Human Subjects’ Protections at Macon & Joan Brock Virginia Health Sciences at Old Dominion University (IRB#: 22-11-FB-0225).

### Donor screening procedures

Per HMBANA Standards, the KDMB employs a comprehensive three-step screening process for all potential donors:

#### Phone interview

Applicants participate in a 20 min telephone interview with a trained staff member, covering topics such as current infant feeding practices, surplus milk availability, milk collection and storage details, and the health history of both the mother and infant. The KDMB interview includes specific drug screening questions such as “In the past 12 months, have you used any street drugs or illicit drugs, including marijuana?” and “In the past 12 months, have you used any prescription medication just for the feeling, more than prescribed, or that were not prescribed for you?” Affirmative answers to these questions require further investigation and may result in a period of milk deferral or disqualification as a donor per HMBANA Standards [12].

#### Electronic questionnaire and healthcare provider communication

Applicants passing verbal phone screening then complete an electronic questionnaire with additional drug screening questions, including “In the past 5 years, have you used recreational drugs such as marijuana, cocaine, LSD, ecstasy, amphetamines, or prescription medications not prescribed for you?” and “In the past 12 months, have you ingested, smoked, or applied CBD or THC products?” Affirmative answers to these questions require further investigation and may result in a period of milk deferral or disqualification as a donor per HMBANA Standards [12]. Additional items also inquire about high-risk behaviors that extend beyond the individual donor to include their household members and sexual partners. KDMB also communicates with both maternal and infant healthcare providers to gather information about maternal health, prenatal serologies, medications, history of substance abuse, and infant health concerns.

#### Serologic testing

Applicants undergo serologic testing for hepatitis B, hepatitis C, HIV, human T-lymphotropic virus, and syphilis. Donors with positive tests are deferred or disqualified per HMBANA Standards [12].

Donor approval is granted only after review of all application documentation by the KDMB milk bank director (BSN, RN, IBCLC) and medical director (MD, IBCLC).

### Milk collection and processing

Breast milk is expressed by donors in their personal environment using either manual or electric pumps. The milk is stored in dated human milk storage bags and promptly refrigerated and frozen within 96 h of expression. Frozen milk is delivered or shipped overnight to the milk bank. For batch processing, milk from 4–8+ approved donors is selected, thawed, pooled, bottled, and labeled. The batches are then pasteurized using the Holder Method (62.5 °C for 30 min) and immediately refrozen. One bottle from each batch is chosen at random and sent to a Clinical Laboratory Improvement Amendments (CLIA)-accredited lab for microbiological culture; any batch testing positive for microbial growth is discarded.

### Sample collection for drug testing

During processing, one bottle of batched milk contains the temperature-sensing probe. This bottle is typically discarded after processing. For this study, a small aliquot of milk was collected from this discard bottle for drug testing. Samples were collected from each batch of pooled, PDHM processed during the study period. The random frozen samples were shipped on dry ice to the InfantRisk Center of Excellence Laboratory at Texas Tech University Health Sciences Center for testing.

### Analytical testing methods

The selection of drug analytes for testing was based on substances with the highest prevalence of use in the local population, as determined through consultations with the state drug laboratory and review of national drug use publications. This targeted approach focused on amphetamines (methamphetamine, MDMA), benzodiazepines (alprazolam), opioids (fentanyl, hydrocodone, hydromorphone, 6-acetyl morphine, morphine, oxycodone, oxymorphone), cocaine and its metabolite benzoylecgonine, and marijuana compounds (Delta 9-THC, 11-OH THC, 11-COOH THC), representing the most common substances of concern for potential exposure in this population. The analytes were obtained from Cerilliant corporation, division of Sigma–Aldrich. Deuterated internal standards for these analytes were also sourced from Cerilliant Corporation. All the standard solutions received were already prepared in solution and were further diluted with methyl alcohol for subsequent method development and analysis.

LC-MS/MS analyses were performed using a Shimadzu LC-40 system connected to an AB Sciex 7500 mass spectrometer system in Multiple Reaction Monitoring (MRM) mode, operating the source in positive ion mode, equipped with an electrospray (ESI) ion source. Table 1 describes the 15 analytes, which were tested, with their respective limits of quantification (LOQ). Chromatographic separation was achieved at 40 °C using a Restek Biphenyl column (100 mm × 2.1 mm i.d., 2.7 μm particle size). The mobile phase consists of solvent A: (2 mM ammonium formate and 0.2% formic acid) and solvent B: (methyl alcohol: Acetonitrile 70:30 v/v, 0.2% formic acid and 2 mM ammonium formate). The flow rate was kept constant at 0.4 mL/min during the analysis, and the sample volume injected was 5 μL, and followed by gradient elution. Acquisition was performed in multiple reaction monitoring (MRM) mode. For sample extraction, 100 μL of milk sample (including blanks, calibrators, QC, and real samples), 10 μL of deuterated internal standard mix (5 ng of each analyte), and 400 μL of acetonitrile were added. The mixture in the tubes was vortexed for 5 min and centrifuged at 14,000 rpm for 10 min at 4 °C temperature. The supernatant was transferred, the organic layer evaporated, and reconstituted in 100 μL of 10% of solvent A as described above. For cannabinoids extraction, 100 μL of human milk was used along with 10 μL of mix of a deuterated internal standard mix (100 ng stock). After adding 300 μL of acetonitrile, mixture was vortexed, centrifuged, supernatant was dried. The final sample was reconstituted in 100 μL of water: methyl alcohol (15:85 v/v).

**Table 1:** Compounds analyzed and their limits of quantification (LOQ).

Compound/Analyte	LOQ in milk (ng/mL)	Samples < LOQ (n (%))
**Amphetamines**		
Methamphetamine	0.3	150 (100%)
MDMA (“ecstasy”)	0.039	150 (100%)
**Benzodiazepines**		
Alprazolam	0.039	150 (100%)
**Opioids**		
Fentanyl	0.078	150 (100%)
Hydrocodone	0.078	150 (100%)
Hydromorphone	0.039	150 (100%)
6-Acetyl Morphine	0.156	150 (100%)
Morphine	0.078	150 (100%)
Oxycodone	0.078	147 (98%)
Oxymorphone	0.3	150 (100%)
**Cocaine**		
Cocaine	0.156	150 (100%)
Benzoylecgonine	0.078	150 (100%)
**Marijuana**		
Delta 9-THC	1.5	150 (100%)
11-OH THC	1.5	150 (100%)
11-COOH THC	3.12	150 (100%)

*LOQ* limit of quantification, *MDMA* 3,4-methylenedioxymethamphetamine, *THC* delta-9-tetrahydrocannabinol, *11-OH THC* 11-hydroxy-delta-9-tetrahydrocannabinol, *11-COOH THC 11-nor-9*-carboxy-delta-9-tetrahydrocannabinol.

Method validation included linearity, limits of detection (LOD) and quantification (LOQ), imprecision, accuracy, selectivity, matrix effect, and recovery. Linearity was determined by least-squares regression with 1/x2 weighting. Calibration curves in blank human milk were validated across a range of 0.039–5 ng/mL for all analytes and their limit of quantification described in Table 2. Linearity was considered acceptable with the coefficient of determination (R2) of at least 0.99. Quality control standards were prepared at low, medium, and high concentrations for all analytes.

**Table 2:** Oxycodone quantification and interpretation in the PDHM samples >LOQ.

Parameter	Sample 1	Sample 2	Sample 3
**Oxycodone concentration** (ng/ml)	0.17	0.425	0.59
**DID** (mg/kg/day)	0.00003	0.00006	0.00009
**RID** (%)	0.006%	0.016%	0.022%
**Comparison to 10% safety threshold**	1563x lower	625x lower	455x lower

*DID* daily infant dose, calculated as [oxycodone] × 200 mL/kg/day, *LOQ* limit of quantification, *PDHM* pasteurized donor human milk, *RID* relative infant dose (%), calculated as DID/therapeutic infant dose × 100.

## Results

Of the 150 PDHM samples tested, 147 (98%) showed no detectable levels of any of the 15 illicit or prescription drug analytes (Table 1). The comprehensive screening panel yielded negative results for all tested amphetamine derivatives (methamphetamine, MDMA), benzodiazepines (alprazolam), most opioids (fentanyl, hydrocodone, hydromorphone, 6-acetyl morphine, morphine, oxymorphone), cocaine and its metabolite benzoylecgonine, and all cannabis-related compounds (Delta 9-THC, 11-OH THC, 11-COOH THC) at their respective limits of quantification. Only three samples (2%) contained trace levels of oxycodone, while all other analytes remained undetected across the entire sample set.

The relative infant dose (RID) for oxycodone was calculated by comparing the estimated infant exposure through milk to a therapeutic infant dose. First, the estimated Daily Infant Dosage (DID) of oxycodone was calculated using the formula: DID (mg/kg/day) = milk concentration (mg/mL) × 200 mL/kg/day. This calculation assumes a standard milk consumption of 200 mL/kg/day for early infancy, as per FDA guidance for evaluating young infant exposure to maternal medications in milk [21]. Then, the relative infant dose (RID) was calculated by comparing the estimated oxycodone exposure through milk to a direct therapeutic infant dose: RID (%) = DID (mg/kg/day)/Infant Therapeutic Oral Dose (mg/kg/day) × 100 [21, 22]. The infant therapeutic dosage used was 0.4 mg/kg/day, in alignment with the 2022 evaluation of oxycodone in human milk used to update the FDA package insert for oxycodone [23]. Using the maximum detected oxycodone concentration in this study, the RID was calculated to be <0.022% of the infant dose as described in Table 2. A RID below 10% has long been accepted as a safe level of infant drug exposure by the World Health Organization and US FDA [22]. The maximum amount of oxycodone detected in milk in this study was 455 times lower than the generally accepted safety threshold of 10%.

## Discussion

This study detected no clinically significant residues of illicit or prescription drugs in 150 samples of infant-ready PDHM, representing 742 individual donor contributions from a HMBANA-accredited milk bank. Screening included 15 analytes, selected based on their anticipated regional prevalence for potential use by lactating individuals, including opioids, amphetamines, cannabis, cocaine, and benzodiazepines.

Trace amounts of oxycodone were detected in 2% of samples, at levels more than 455 times below established safety thresholds that pose no clinical risk to even medically fragile neonates. Despite using highly sensitive detection methods with limits of quantification well below standard clinical urine screening thresholds, no cannabinoid residues were detected in any sample. The absence of detectable levels of marijuana compounds is particularly noteworthy given that cannabis is reported to be the most commonly used drug among breastfeeding individuals.

Our findings are consistent with other studies validating the effectiveness of rigorous screening in mitigating the risks of illicit and prescription drug residues in PDHM [11, 16, 17]. Our study extends this work by testing for a broader range of drugs and their metabolites, including commonly prescribed opioids with known abuse potential, using sensitive analytical methods (LC-MS/MS).

Though it is one of the most commonly abused opioids in our local community, oxycodone is also one of the most commonly prescribed medications for postpartum pain management [24], and the traces detected likely represent residue persistence following legitimate therapeutic use. The pharmacokinetics of oxycodone—with its approximately 4 h elimination half-life in adults—would suggest complete clearance within 24 h (representing the standard FDA washout period of six half-lives). KDMB implements a highly conservative deferral period for donations following oxycodone administration, exceeding a standard FDA washout period. However, modern highly sensitive LC-MS/MS methodology can still detect sub-therapeutic concentrations long after clinically meaningful exposure has ceased. This detection phenomenon is not unique to human milk; environmental studies have documented oxycodone at concentrations of 0.005 parts per billion (ppb) in US wastewater [25], with effluent near pharmaceutical manufacturing facilities reaching significantly higher concentrations of 1700 ppb [26].

The oxycodone levels detected in this study can be further contextualized using pharmacokinetic data from the NICHD-2017-BMS01 study on oxycodone in lactating women and their breastfed infants, which reported maternal milk concentrations reaching up to 168 ppb in those taking therapeutic doses [23]. After analyzing infant serum levels, the report concluded that maternal oxycodone doses totaling below 60 mg/day are unlikely to result in clinically relevant exposures for breastfed neonates—a conclusion that the FDA has endorsed, leading to updates in the drug’s approved labeling [27]. The trace amounts of oxycodone in our pooled donor milk samples, less than 0.6 ng/ml (0.6 ppb), represent an even lower exposure risk.

The results of this study support the effectiveness of the comprehensive donor screening and processing practices employed by a HMBANA-accredited milk bank. The multi-stage approach—combining detailed interviews, questionnaires, healthcare provider communication, and serologic testing—appears to successfully identify, exclude, and/or mitigate the risk of donors who might introduce drugs into the milk supply at meaningful levels. The voluntary, non-remunerated donation model likely contributes to this safety profile by attracting motivated, altruistic donors who are committed to infant health and willing to comply with rigorous screening procedures. This contrasts with ethical concerns about commercial models, where financial compensation might incentivize non-disclosure of disqualifying behaviors. Additionally, two processing practices help mitigate the risk of harmful substances in processed donor milk: combining milk from different pumping dates—given that individual donor deposits typically span several weeks to months—and pooling milk from multiple donors. Pooling not only enhances the nutritional consistency and bioactive diversity of the final product but also increases safety by diluting the potential impact of any single donor’s contribution.

This study has several limitations. First, it examined samples from only one milk bank, and practices may vary slightly between institutions. Second, despite the substantial sample size (*n* = 150) of pooled milk, rare occurrences might not be captured. Third, the study was limited to 15 specific drug analytes, though these represent the most common drugs of concern. Lastly, our consent language prevented the tracing of positive detections in pooled PDHM batches back to individual donors, which limited our ability to conduct follow-up investigations on the source and circumstances of contaminant introduction.

A major strength of this study is its use of rigorous analytical methodology. The LC-MS/MS method employed detection thresholds substantially below standard clinical screening levels (e.g., cannabinoid LOQs of 1.5–3.12 ng/mL compared to the 50 ng/mL threshold used in standard urine drug tests). The representative sampling across an 18-month timeframe provides confidence that the findings reflect typical operations rather than a limited snapshot. Additionally, the study design evaluated the actual product provided to infants (pasteurized, pooled milk), enhancing the clinical relevance of the findings.

### Implications for practice

These findings support the current screening and processing practices employed by a HMBANA-accredited milk bank. The comprehensive donor screening process appears effective in preventing clinically significant illicit and prescription drug exposure through PDHM.

The results, in addition to similar studies, suggest that routine analytical testing for drugs of abuse in PDHM may not be necessary given the effectiveness of current screening protocols. This has important implications, as such testing is expensive and logistically challenging, with no FDA-approved in vitro diagnostic tests available for a human milk matrix.

For clinicians, these findings provide reassurance about the safety of properly screened donor milk from HMBANA-accredited milk banks with respect to drug exposure, supporting its continued use for vulnerable infants who cannot receive mother’s own milk.

Future research should expand the range of tested drug analytes, increase sample size, incorporate samples from additional milk banks, and obtain consent for retrospective chart reviews to investigate the etiology of positive samples.

## Conclusion

This study found no clinically significant illicit or prescription drug residues in pasteurized donor human milk from a HMBANA-accredited milk bank, supporting the safety and effectiveness of current donor screening and processing practices. The trace amounts of oxycodone detected in a small percentage of samples were at levels more than 455 times below established standards for infant safety posing no clinical risk to infants, (Relative Infant Dose: <0.022%; standard safety threshold 10%).

These findings provide reassurance to healthcare providers and families about the safety of properly screened and processed donor human milk from not-for-profit milk banks with respect to potential drug exposures. The results support the continued use of comprehensive screening protocols rather than routine analytical testing for drugs of abuse in donor milk.

## Data Availability

The datasets used and analyzed during the current study are not publicly available due to institutional/ethical/proprietary restrictions, but may be available from the corresponding author upon reasonable request and with appropriate approvals.
